# Antinociceptive effects of a hydroethanolic stem bark extract of *Burkea africana*

**DOI:** 10.1016/j.heliyon.2022.e08917

**Published:** 2022-02-09

**Authors:** Yakubu Jibira, Eric Boakye-Gyasi, Wonder Kofi Mensah Abotsi, Isaac Kingsley Amponsah, Peter Duah, Frederick Kwadwo Baah, Eric Woode

**Affiliations:** a Department of Pharmacology, Faculty of Pharmacy and Pharmaceutical Sciences, Kwame Nkrumah University of Science and Technology (KNUST), Kumasi, Ghana; b Department of Pharmacognosy, Faculty of Pharmacy and Pharmaceutical Sciences, Kwame Nkrumah University of Science and Technology (KNUST), Kumasi, Ghana; c Department of Pharmacology, University of Health and Allied Sciences, Ho, Volta Region, Ghana; d Department of Pharmacology and Toxicology, University for Development Studies, Tamale, Northern Region, Ghana

**Keywords:** *Burkea africana*, Acidic-saline, Acetic acid, Hyperalgesia, Formalin

## Abstract

**Introduction:**

Pain is a major symptom of many clinical disorders and its relief has long been a concern for individuals across the globe. There is therefore an unmet need to search for new efficacious agents for the effective management of pain. The stem bark of the savanna tree *Burkea africana* (Hook) (Family: Leguminosae) is used in the Ghanaian traditional medicine for the treatment and management of various pain-related diseases.

**Method:**

An acute oral toxicity study in mice was conducted by administering BAE (50–5000 mg kg^−1^*p.o*.). Antinociceptive effect of BAE (50–1000 mg kg^−1^*p.o*.) was evaluated using the acetic acid-induced abdominal constriction, acidic saline-induced muscle pain and formalin-induced pain models. The antinociceptive mechanism of BAE was also assessed using the formalin-induced pain model.

**Results:**

The LD_50_ of BAE was thus estimated to be above 5000 mg kg^−1^ since none of the animals died in the acute toxicity study. Pretreatment with BAE (50–1000 mg kg^−1^*p.o*.) significantly reduced the number of writhes after acetic-acid administration compared to the vehicle treated group. BAE also produced a significant and dose-dependent reversal of mechanical hyperalgesia induced by the injection of the acidic saline. Administration of BAE was able to significantly suppress both phases of the formalin test. This effect of the extract was however reversed by pretreatment with naloxone and granisetron.

**Conclusions:**

BAE exhibits antinociceptive effects in rodent pain models with a possible involvement of 5-HT_3_ receptors and opioidergic pathways.

## Highlights

• *Burkea africana* stem bark extract.
• Acetic acid-induced abdominal constriction.
• Acidic saline-induced muscle pain.
• Formalin-induced pain.

## Graphical abstract

**Figure:**
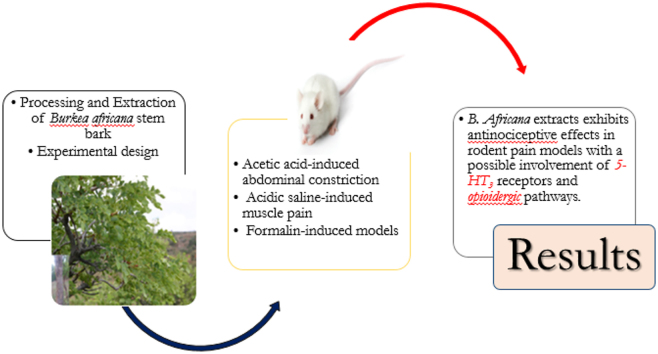

## 1 Introduction

Pain is associated with virtually all clinical diseases and is mostly the principal symptom that prompt patients to seek medical attention [1]. It represents a wide clinical and socio-economic problem across all age groups [2]. The effect of pain on economies is huge, with the total estimated cost of pain evaluated to be up to 3.0 % of the Gross domestic product (GDP) of the world [2]. The yearly estimated cost of pain management is more than the cost incurred for cardiovascular illness or cancer across the globe [3]. In developing countries including Ghana, about 20 % of the adult populace endure the menace of pain due to poor management or lack effective treatment regimen [2]. When there is a tissue damage due to mechanical, thermal or chemical stimuli, it's followed by a release of pro-nociceptive substances and activation of the noxious receptors at the terminal ends of the peripheral nerves. The pro-nociceptive substances includes serotonin, noradrenaline, histamine, enkephalins, beta-endorphins, dinorphins, acetylcholine, glutamate, Gamma-aminobutyric acid (GABA), Nerve Growth Factor (NGF) and calcitonin gene-related peptide (CGR), tachykinins, substance P, bradykinin, prostaglandins (E and F) and lactic acid, Adenosine triphosphate (ATP), Adenosine diphosphate (ADP), potassium ion [4, 5, 6].

Drugs currently used in pain management include non-opioid agents (e.g. steroidal and non-steroidal anti-inflammatory drugs, disease modifying anti-rheumatic drugs, antidepressants and anticonvulsants) and the opioids. Most of these existing analgesics are relatively ineffective for the management of chronic pain and also their persistent use comes with the burden of side effects or potential for drug abuse [7].

The use of plants extracts and phytochemicals both with known pharmacological effects can be of great importance in the management of pain. In the past decades, several studies have been conducted by numerous researchers across the globe to screen several medicinal plants in an attempt to add scientific backing to their use as analgesic in folk medicine [8, 9, 10, 11].

Medicinal plants present an enormous repository of potential leads for the development of novel pharmacological agents. One such medicinal plant is *Burkea africana,* used traditionally to manage pain in some rural communities of the northern part of Ghana [12, 13]. In Ghana an aqueous preparation of the root in employed in treating conjunctivitis, edema, stomach pain and toothaches [14]. It is also used together with other plant materials for treating various forms of pain. This present study therefore sought to validate the possible antinociceptive effects of the hydroethanolic stem bark extract of *Burkea africana* as suggested by folklore medicine and elucidate some of the probable mechanisms involved in its actions.

## 2 Materials and methods

### 2.1 Chemicals and reagents

Theophylline anhydrous, Glibenclamide, Nifedipine (Sigma Aldrich, St-Louis, MO, USA), Morphine sulfate (Hameln pharmaceutical Ltd, Gloucester, UK), Diclofenac sodium (Diclowin®) (Hubei Tianyao Pharmaceutical Co. Ltd, Huanggang, China), Naloxone hydrochloride, Reserpine (SG-Pharma, Mumbai, Maharashtra, India), Yohimbine hydrochloride (Akorn Pharmaceutical, Decatur, IL), Granisetron hydrochloride (Actiza Pharmaceutical Pvt. Ltd., Gujarat, India).

### 2.2 Animals

ICR mice (30 ± 5 g) used in this study were purchased from the Center for Scientific Research into Plant Medicine (CSRPM) at Mampong-Akuapem in the Eastern region Ghana and the Sprague Dawley rats (200 ± 5 g) were also obtained from Noguchi Memorial Institute for Medical Research (NMIMR), Legon, Greater Accra region, Ghana. The mice and rats were habituated at the Department of Pharmacology, KNUST, *vivarium*. The animals were randomly grouped (n = 10 for mice and n = 5 for rats) and kept in stainless steel cages with 34 × 47 × 18 cm^3^dimension. The housing environment was maintained at a temperature of 26 ± 0.5 °C, relative humidity of 65 ± 5% in a 12 h day and night cycle and provided water *ad libitum.*

All the experimental designs conform to the Department of Pharmacology Ethics Committee standards and the Guide for the Care and Use of Laboratory Animals, 8th edition (Number: 407), the National Institute of Health Guidelines for the Care and Use of Laboratory Animals (NIH, Department of Health Services publication No. 83-23, revised 1985). All individuals involved in the experimental study observed all institutional biosafety guidelines for protection of personnel and laboratory. The animals were trained to acclimatize to working environment before the start of each experiment.

### 2.3 Collection and extraction of plant material

Matured stem bark of *Burkea africana* was harvested from Tamale in the Northern region of Ghana in April, 2017 at a Latitude of 9° 59′ 29.6797″ N and a Longitude of 2° 30′ 51.5059″ W. The stem bark was identified and authenticated at the department of Herbal Medicine, Faculty of Pharmacy and Pharmaceutical sciences, KNUST, Kumasi, Ghana by Dr. George Henry Sam. The sample was pressed and kept at the faculty's herbarium with the voucher number: KNUST/HM1/2017/SB005.

The fresh plant material was cleaned and air dried for 120 h. The dried plant material was milled into a coarse powder electronically (Hammer mill, Christy and Norris, Chelmsford, England). The powdered bark was extracted using a Soxhlet with hydro-ethanol. The liquid extract was processed using a rotary evaporator (Rotavapor R-215, BÜCHI Labortechnik AG, Flawil, Switzerland) to yield a semi-solid mass and dried using an electric oven (Leader Engineering, Widnes Cheshire, UK) at 35 °C. The final yield of the hydroethanolic extract of *B. africana*, denoted as BAE, was 10.85 %^w^/_w_.

### 2.4 Phytochemical screening

The qualitative phytochemical evaluation was carried out on the dried powder of *B. africana* stem bark using standard methods as described by Prashant *et al.*, [15].

### 2.5 Test for alkaloids

1 ​ml of the 1% HCl extract solution was treated with a few drops of Dragendoff's reagent and an orange-red precipitate formed shows the presence of alkaloids.

### 2.6 General test for glycosides (reducing sugar test)

About 10 mg of the extract was heated over the water bath with dilute H_2_SO_4_ for 5 min. It was then filtered and about 2–10 drops of 20% NaOH was added to make the filtrate completely alkaline. Fehling's solution A and B were added and heated on the water-bath for about 2 min. A brick-red precipitate formed shows the presence of glycosides.

### 2.7 Test for tannins

The extract was treated with distilled and filtered, then 1% gelatin solution containing sodium chloride was added to about 1 ml of the filtrate. The formation of a white precipitate shows the presence of tannins.

### 2.8 Test for flavonoids

Aqueous solution the extract was treated with 5–10 drops of sodium hydroxide solution. And the forming of intense yellow color, which turns colorless following the addition of drops of dilute acid, shows that flavonoids are present.

### 2.9 Test for saponins (the foam's test)

About 0.25 % wv extract solution was shaken vigorously. The formation of a persistent froth for 10 min shows the presence of saponins.

### 2.10 Test for triterpenes (Salkowski's test)

Chloroformic solution of the extract was prepared and the filtrate treated with few drops H_2_SO_4_ and allowed to stand after shaking. A golden yellow ring at the interface indicates the presence of triterpenes.

### 2.11 Test for phytosterols (Liebermann Burchard's test)

The extract was dissolved in chloroform and filtered. The filtrate was treated with few drops of acetic anhydride, warmed on a water bath. After cooling a few drops of H_2_SO_4_ was added and the formation of brown ring at the junction between the interface and the chloroform layer shows phytosterols are present.

### 2.12 Acute oral toxicity test

Twenty-four ICR male mice were divided into four groups (n = 6). Prior to the investigation, the animals were deprived of food for 3 h. Group 1 served as the vehicle control group and received normal saline orally. Groups 2, 3 & 4 received BAE 50, 500 and 5000 mg kg^−1^ *p.o*. The animals were then monitored continuously for every 30 min over a 24 h period to observe for changes in morphological, behavioral, neurological and autonomic responses or death. The experimental protocol and procedure used was in accordance with OECD guidelines for testing chemicals acute oral toxicity [16].

### 2.13 Antinociceptive tests

#### 2.13.1 Acetic acid-induced abdominal constriction model

The acetic acid-induced writhing test was carried out as described elsewhere [17, 18]. Mice were randomly grouped (n = 5) and treated with either 10 mL kg^−1^ of 0.9 % wv normal saline, i.p., BAE (50, 500 & 1000 mg kg^−1^, *p.o.*), diclofenac (10, 30 and 100 mg kg^−1^, i.p.) or morphine (1, 3 and 10 mg kg^−1^, i.p.). Each animal was injected with 0.6 % acetic acid intraperitoneally and placed in a glass chamber (15 × 15 × 15 cm) with a mirror inclined at 45^o^ underneath the transparent floor of the chamber. Abdominal contractions together with the stretching of the hind limbs were recorded for 30 min using a digital camera (Camera Maker-Olympus Imaging, model, Japan) clamped directly opposite the mirror. The total abdominal contractions and stretching were quantified using the software JWatcher™, Version 1.0 (University of California, LA, U.S.A. and Macquire University, Australia) to obtain the frequency and duration of the abdominal contractions together with the hind limbs stretching per 20 min.

#### 2.13.2 Acidic saline-induced muscle pain model

This test was carried out as described by Sluka *et al* [19]. Rats (20) were anesthetized using sodium pentobarbitone (40 mg kg^−1^, i.p.). The gastrocnemius muscle of the left hind limb was injected aseptically with 0.1 mL of acidic saline (pH 3.5). Five days after the first injection, rats were re-anesthetized and the same muscle injected with the same volume of acidic saline. Hyperalgesia was measured with the Randall Sellito method as described by Woode *et al.* [20], a day before the first acidic saline injection and 24 h after the second injections to establish the presence of hyperalgesia. Three hours after the establishment of hyperalgesia, rats were treated with either vehicle (10 mL kg^−1^ of 0.9% NaCl, i.p.), BAE (50, 500 and 1000 mg kg^−1^, p.o.), morphine (1, 3 and 10 mg kg^−1^, i.p.) or diclofenac (10, 30 and 100 mg kg^−1^, i.p.). Readings were taken every hour up to the 32 h of the second acidic saline injection.

#### 2.13.3 Formalin-induced pain model

The formalin test was carried out as described elsewhere [20, 21]. Groups of mice (n = 5) were pretreated with the vehicle (10 mL kg^−1^ of normal saline, i.p.), BAE (50, 500 and 1000 mg kg^−1^, *p.o*.) or morphine (1, 3 and 10 mgkg^-1^, i.p.) half an hour for the intraperitoneal and an hour for the oral before 10 μL of 5 % formalin was injected into the left foot-pad. After the formalin injection, each mouse was instantly transferred into transparent testing perspex chambers (15 cm × 15 cm × 15 cm). A mirror placed at 45° to the floor level allowed complete view of the animals in the digital camera (Camera Maker-Olympus Imaging, model, Japan) which was used to capture the nociceptive behaviors of the mice following formalin injection.

In a second formalin test, groups of mice (n = 5) were pretreated with different antagonists; naloxone (2 mg kg^−1^ *i.p*.), theophylline (5 mg kg^−1^ *i.p.*), glibenclamide (8 mg kg^−1^ *p.o.*), yohimbine (3 mg kg^−1^ *p.o.*), granisetron (2 mg kg^−1^ *i.p.*), reserpine (5 mg kg^−1^ *i.p.*) or nifedipine (10 mg kg^−1^ *p.o.*) (1/2 h for *i.p.* and 1 h for *p.o*) before the administration of either BAE (500 mg kg^−1^, *o.p.*) or morphine (3 mg kg^−1^, *i.p.*). Doses of antagonists were selected based on preliminary studies and also from literature [22]. 10 μL of 5 % formalin was injected into the left foot-pad of mice 1 h after BAE and 30 min after morphine administration.

Each formalin test was recorded for 60 min and later tracked using a JWatcherTM software Version 1.0 developed by Macquarie University, Sydney, Australia and University of California, Los Angeles, USA. A nociceptive score for every 5 min time block was obtained by measuring the frequency and duration of licking/biting of injected paws. Average nociceptive score for each time block was calculated as the product of the duration and frequency of licking/biting. The results obtained were considered as first/neurogenic phase (0–10 min) and second/inflammatory phase (10–60 min).

### 2.14 Statistics

In this study, the ordinary two-way (*treatment against time*) analysis of variance (ANOVA) followed by the Dunnett's comparison test used to statistically compare the *treatment* (between subjects) and the *time* (within subjects) as factors verses the average treatment effects at the various time period. The overall nociceptive score for the individual treated groups was computed as the area under the curves (AUC). In this study, the changes in treatment outcomes compared to the non-treated group were expressed using the mathematical formula below:

(1) $$Percentage\;Inhibition=\left|\frac{AUC_{control}-AUC_{treatment}}{AUC_{control}}\right|x100\%$$

The difference in total anti-nociceptive score was determined using one-way ANOVA with Turkey's *post hoc* test using treatment data as the between-subject factor for data which were distributed normally. Kruskal-Wallis test with Dunn's *post hoc* test was used for evaluating differences in total anti-nociceptive effect of the data that were not normally distributed.

Doses responsible for half of the highest effect (ED_50_) for the various drug was evaluated using a repetitive graph following non-linear regression (three-parameter logistic) formula:

(2) $$Y=\frac{a+\left|b-a\right|}{\left|1+10^{\left|Log\;ED_{50}-X\right|}\right|}$$

where: X represents the logarithm of dose used in the study and Y symbolizes the response. Y starts from the bottom, (a) and goes to the top (b) of the sigmoid shape. *F* test was used to statistically compare the fitted midpoints (ED_50_S) of the curves.

## 3 Results

### 3.1 Phytochemical screening

The qualitative phytochemical tests revealed the presence of alkaloids, flavonoids, saponins, tannins, reducing sugars, phytosterols and terpenoids (Table 1).

**Table 1:** Phytochemical constituents of the ethanolic stem bark extract of *B. africana*.

Secondary metabolite	Inference
Alkaloids	**+**
Reducing sugars	+
Tannins	+
Flavonoids	+
Saponins	**+**
Triterpenoids	**+**
Phytosterols	**+**

Key: + denotes detected.

### 3.2 Acute toxicity

All the animals survived throughout the 24-h study period and from the observations. There were no behavioral changes in the mice as well as no signs of neurological and autonomic toxicity. The LD_50_ of BAE was thus estimated to be above 5000 mg kg^−1^.

### 3.3 Anti-nociceptive tests

#### 3.3.1 Acetic acid induced abdominal constriction

**Figure 1:**
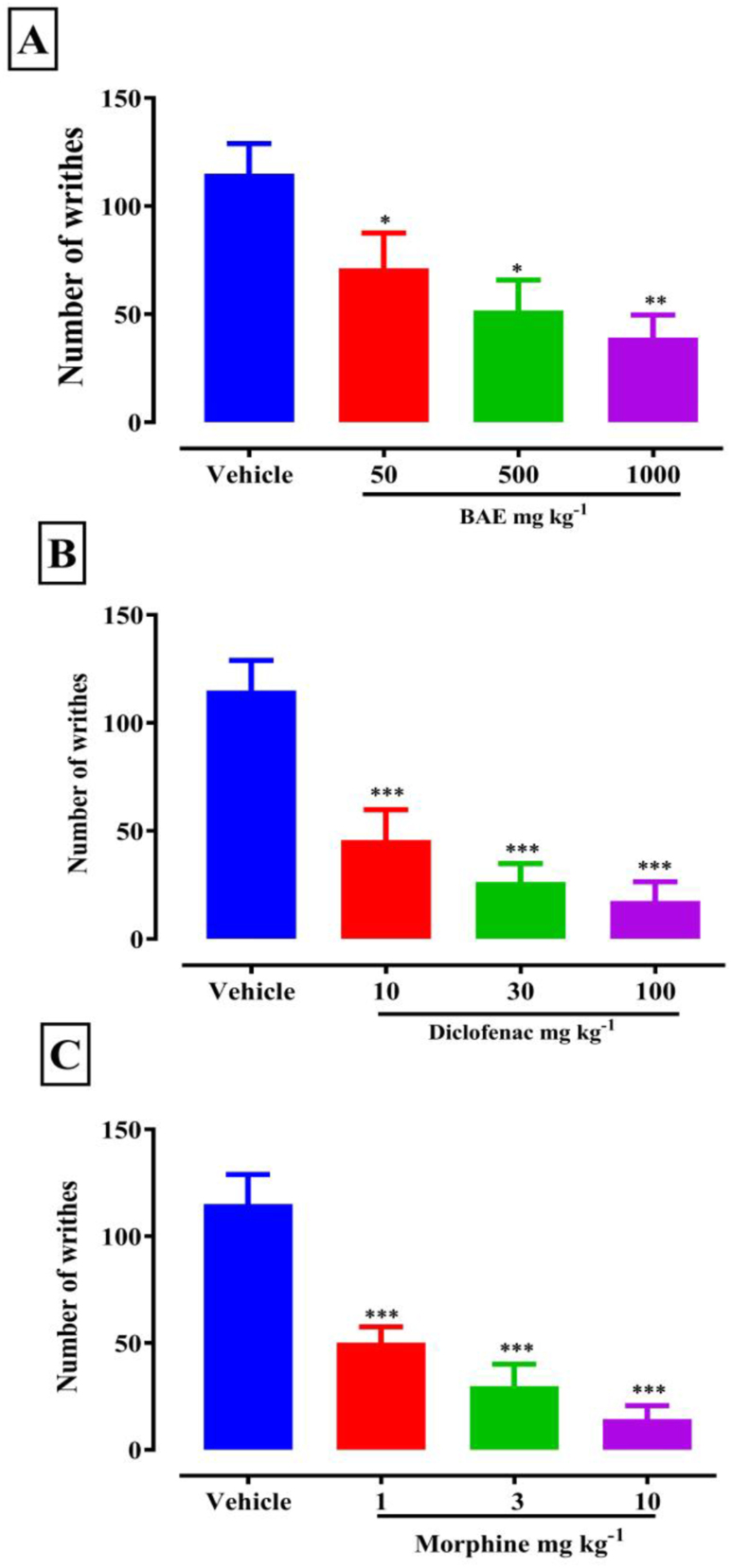
Effect of BAE (50–1000 mg kg^−1^), diclofenac (10–100 mg kg^−1^) and morphine (1–10 mg kg^−1^) on the abdominal constrictions produced by the intra-peritoneal injection of acetic acid in mice; Data are presented as mean ± S.E.M. (n = 5). †P < 0.05; ††P < 0.01; †††P < 0.001 compared to vehicle-treated group (One-way ANOVA followed by Dunnet's test).

BAE (50–1000 mg kg^−1^, *p.o*.), morphine (1, 3 and 10 mg kg^−1,^ *i.p.*) and diclofenac (10, 30 and 100 mg kg^−1^, *i.p*.) significantly (BAE: *F*_(3, 16)_ = 8.34; P = 0.0014, diclofenac: *F*_(3, 16) =_ 14.36; P < 0.0001 and morphine *F*_(3, 16)_ = 19.88; P < 0.0001) (Figure 1) reduced the total number of writhes following acetic-acid administration. BAE (1000 mg kg^−1^) gave a maximal inhibition [Eq. (1)] of 64.31 ± 10.64% (Figure 1a) Similarly, diclofenac (100 mg kg^−1^) and morphine (10 mg kg^−1^) also inhibited the acetic acid-induced abdominal constrictions with maximal inhibitions of 88.89 ± 10.19 and 92.31 ± 8.33 (Figure 1b & c) respectively. From the ED_50_ calculated [Eq. (2)] in Table 2, morphine was the most potent followed by diclofenac and then the extract.

**Table 2:** The ED_50_S of drugs used in the acetic acid- and formalin-induced models [Eq. (2)].

Models	ED_50_S (mg kg^−1^)		
	BAE	Morphine	Diclofenac
Acetic acid-induced writhing	39.69 ± 26.27	0.66 ± 0.29	4.74 ± 4.38
Acidic-saline induced model	30.01 ± 225.77	5.64 ± 10.85	6.99 ± 18.47
Formalin-induced nociception (phase 1)	90.02 ± 32.98	0.63 ± 0.21	-
Formalin-induced nociception (phase 2)	49.94 ± 26.85	2.132 ± 2.06	-

-: diclofenac was not used in the formalin-induced nociception model.

#### 3.3.2 Acidic saline-induced muscle pain

**Figure 2:**
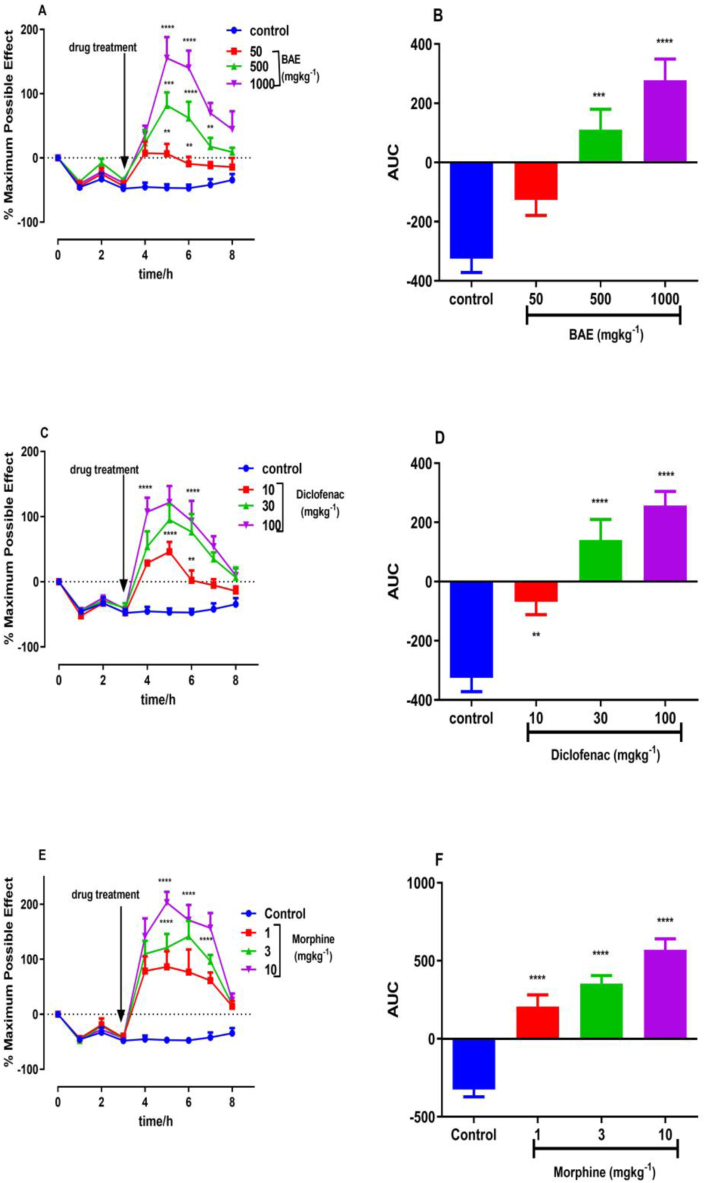
Effect of BAE (50, 500 and 1000 mg kg^−1^), diclofenac (10, 30 and 100 mg kg^−1^) and morphine (1, 3 and 10 mg kg^−1^) on the time course curves (A, C, E) and the AUCs (B, D, F) of acidic acid induced hypernociception in rats. Data are presented as mean ± S.E.M. (n = 5). ∗∗P < 0.01; ∗∗∗P < 0.0001, ∗∗∗∗P < 0.0001 compared to vehicle-treated group (Two-way ANOVA followed by Dunnett's multiple comparison test); ††P < 0.01; †††P < 0.001, ††††P < 0.0001 compared to vehicle-treated group (One-way ANOVA followed by Dunnett's multiple comparison test).

Intramuscular injection of two cycles of a low pH saline solution into the gastrocnemius muscle of the rats produced a non-inflammatory bilateral muscle mechanical hyperalgesia which was sustained for up to 32 h after the second injection (Figure 2 A, C, E).

The paw withdrawal threshold (PWT) increased significant in all drug-treated groups [Two-way ANOVA (treatment × time); BAE*: F*_3, 141_ = 63.25, *P* < 0.0001; Morphine: *F*_3, 141_ = 67.60, *P* < 0.0001; Diclofenac: *F*_3, 140_ = 59.03, *P* < 0.0001; Figure 2A, C, E]. From analysis of the AUCs obtained from the time course curves, BAE (50–1000 mg/kg, *p.o.*) significantly (*F*_3, 16_ = 18.96, *P* < 0.0001; Figure 2B) reversed mechanical hyperalgesia with maximum inhibition [Eq. (1)] of 197.7 ± 51.81% at 10000 mg/kg. Diclofenac (10–100 mg kg^−1^, i.p.) and morphine (1–10 mg kg^−1^, i.p.) also significantly (diclofenac: *F*_3, 16_ = 23.18; *P* < 0.0001; morphine: *F*_3, 16_ = 37.13; *P* < 0.0001; Figure 2D, F) and dose-dependently inhibited the mechanical hyperalgesia. The extract (ED_50_ = 30.01 ± 225.77 mg kg^−1^) was however less potent than morphine (ED_50_ = 5.64 ± 10.85 mg kg^−1^) and diclofenac (ED_50_ = 6.99 ± 18.47 mg kg^−1^) (Table 2) [Eq. (2)].

### 3.4 Formalin-induced pain

**Figure 3:**
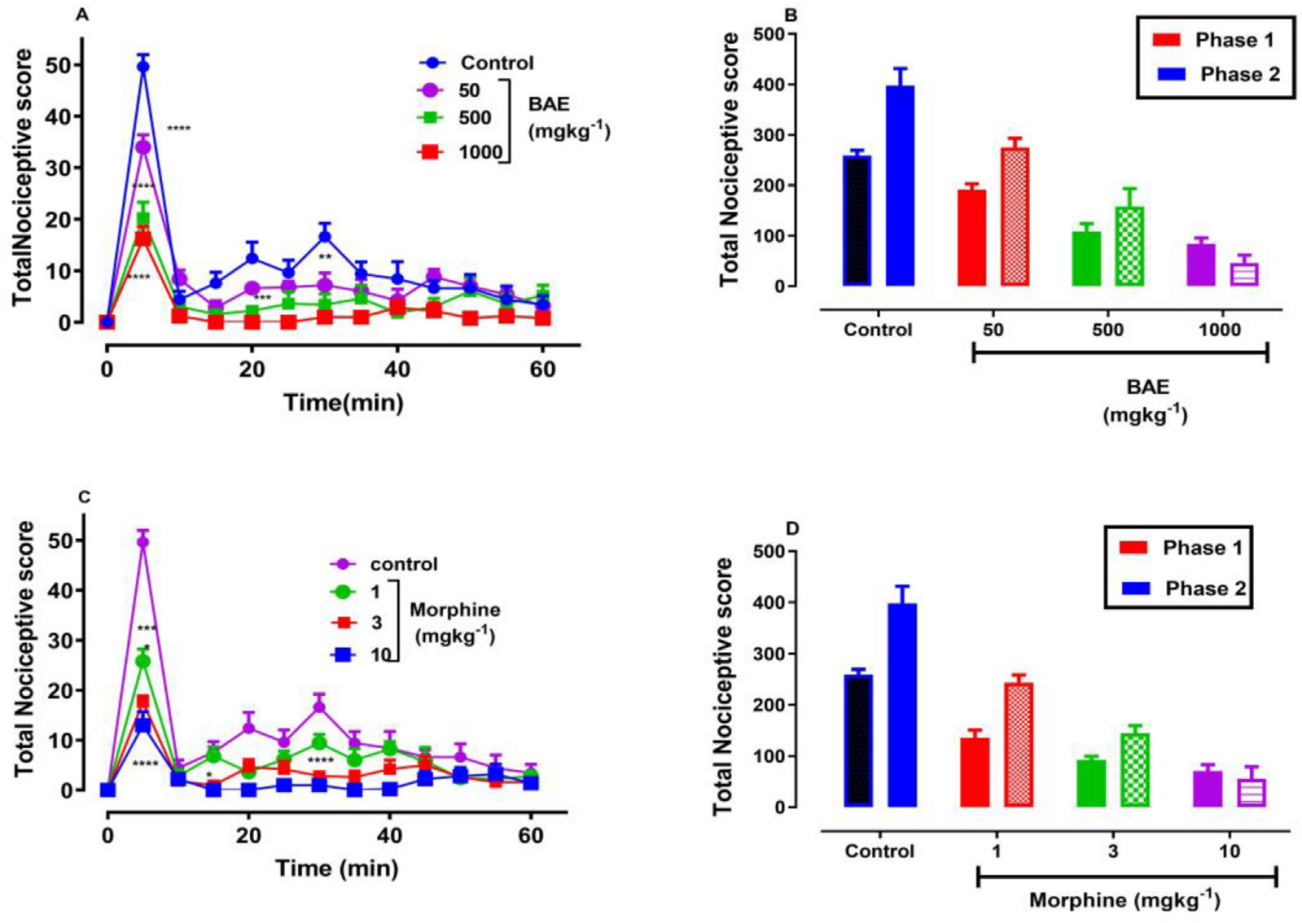
Effect of BAE (50, 500 and 1000 mg kg^−1^) and Morphine (1, 3 and 10 mg kg^−1^) on the time course curve (A, C) and the total nociceptive score (AUCs) (B, D) of formalin-induced nociception in mice. Data are presented as mean ± S.E.M. (n = 5). ∗P < 0.05; ∗∗P < 0.01; ∗∗∗P < 0.001, ∗∗∗∗P < 0.0001 compared to vehicle-treated group (Two-way ANOVA followed by Dunnet's multiple comparison test). ^†^P < 0.05, ^††^P < 0.01; ^†††^P < 0.001, ^††††^P < 0.0001 compared to vehicle-treated group (One-way ANOVA followed by Dunnet's multiple comparison test).

The intraplantar injection of 10 μl of 5 % formalin into the hind paw induced the characteristic biphasic response: an initial neurogenic phase (0–10 min after injection, Figure 3A, C) and a late inflammatory response phase (10–60 min after injection, Figure 3A, C). Drug treatment caused a general inhibition of formalin-evoked nociception compared to vehicle control group (BAE: F_3, 207_ = 54.11, P < 0.0001; morphine: F_3, 208_ = 61.69, P < 0.0001; Two-way ANOVA (treatment x time); Figure 3A, C). One-way ANOVA of AUCs from the time course curves reveal that BAE significantly inhibited the first phase (F_3, 16_ = 41.93, P < 0.0001) and the second phase (F_3, 16_ = 30.67, P < 0.0001), with respective maximal inhibitions of 71.48 ± 5.55% and 66.20 ± 22.28 % [Eq. (1)]. Morphine (1, - 10 mg kg^−1^) also dose-dependently inhibited both the first phase (F_3, 16_ = 52.83; P < 0.0001) and the second phase (F_3, 16_ = 39.51, P < 0.0001), with maximal inhibitions of 77.48 ± 5.33 % and 102.50 ± 14.27 % respectively (Figure 3D). ED_50_s obtained from the non-linear regression curve revealed morphine was more potent in both phases than BAE [Eq. (2)]. Also, BAE was more potent in the second phase than the first phase of the formalin test (Table 2).

### 3.5 Possible mechanism involved in the anti-nociceptive effects of BAE

**Figure 4:**
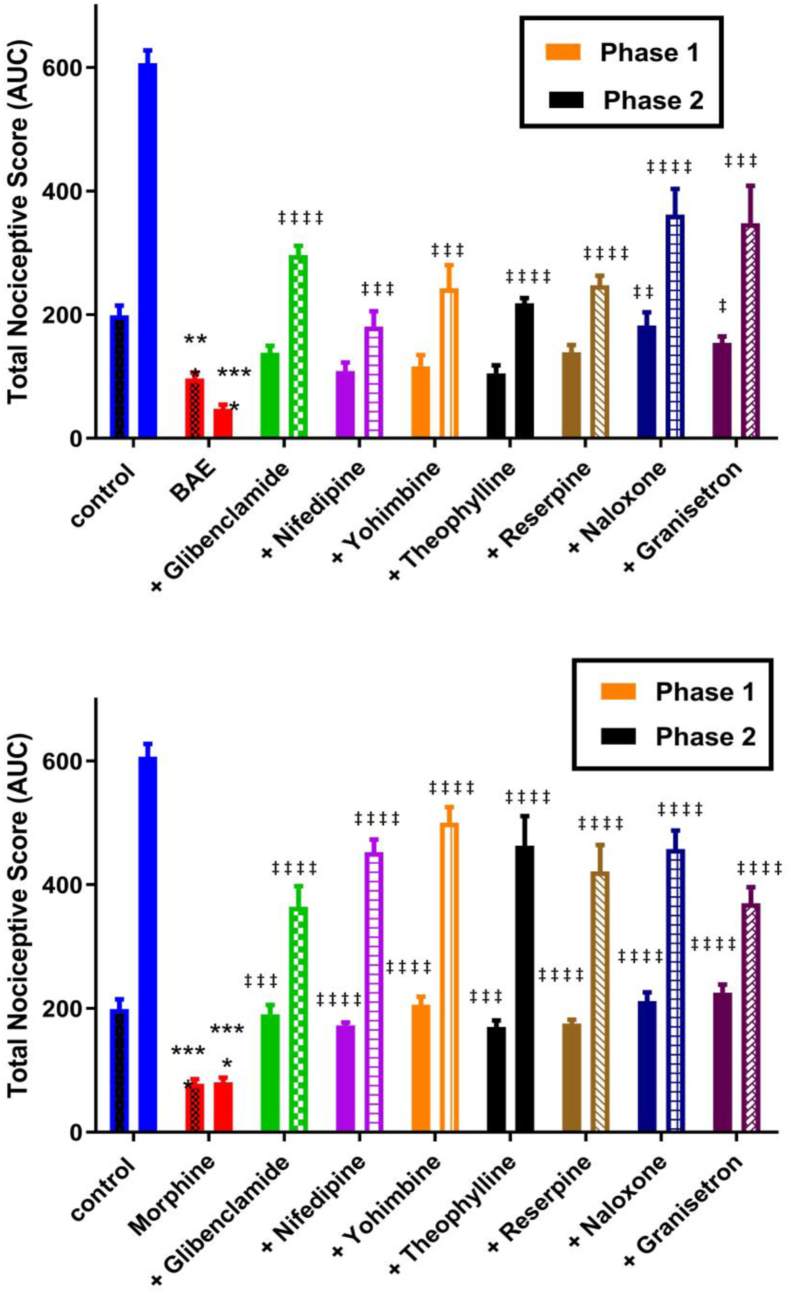
Effect of glibenclamide (8 mg kg^−1^*p.o.*), nifedipine (10 mg kg^−1^*p.o.*), yohimbine (3 mg kg^−1^*p.o.*), theophylline (5 mg kg^−1^*i.p.*), reserpine (5 mg kg^−1^*i.p.*), naloxone (2 mg kg^−1^*i.p*.) or granisetron (2 mg kg^−1^*i.p.*) on the antinociceptive effect of BAE (500 mg kg^−1^*p.o.*) (A) and morphine (3 mg kg^−1^*i.p.*) (B) in both phases of the formalin test. Data are presented as mean ± S.E.M. (n = 5). ∗∗∗*P* < 0.001; ∗∗∗∗*P* < 0.0001 compared to vehicle-treated group (One-way ANOVA followed by Dunnetts's multiple comparison test). ^†^*P* < 0.05; ^††^*P* < 0.01; ^†††^*P* < 0.00, ^††††^*P* < 0.0001 compared to respective drug-treated group (One-way ANOVA followed by Dunnets's multiple comparison test).

Effect of pre-treatment of mice with various antagonists on the antinociceptive activity of BAE and morphine are shown in Figure 4. Pre-treatment of mice with naloxone (2 mg kg^−1^, *i.p.*) or granisetron (2 mg kg^−1^, *i.p*) reversed the antinociception caused by BAE (500 mg kg^−1^, *p.o*.) in both neurogenic and inflammatory phases (Figure 4A). However, pre-treatment with glibenclamie (8 mg kg^−1^, *p.o*), nifedifine (10 mg kg^−1^, *p.o*), yohimbine (3 mg kg^−1^, *p.o*), theophylline (5 mg kg^−1^, *i.p*) or reserpine (5 mg kg^−1^ *i.p.*) could not abolish the antinociceptive effects of BAE in the neurogenic phase but caused a partial reversal in the inflammatory phase (Figure 4A). Previous treatment of mice with naloxone, granisetron, glibenclamie, nifedifine, yohimbine, theophylline or reserpine abolished the antinociception caused by morphine (3 mg kg^−1^, *i.p*) in both phases of the formalin test (Figure 4B).

## 4 Discussion

Preliminary phytochemical screening of the ethanolic extract of the stem bark of *Burkea africana* revealed the presence of alkaloids, saponins, tannins, phytosterols, triterpenoids, reducing sugars and flavonoids. This agrees with a previous report by Yaro *et al* [28]. Plant constituents such as flavonoids, saponins, terpenes and tannins have exerted influence in drug discovery and development research due to their inherent pharmacological prospect [29]. Even though most secondary metabolites in *B. africana* have not been isolated and pharmacologically studied, it is very possible that the alkaloids and flavonoids detected may play a role in the antinociceptive effects and other medicinal usefulness of *B. africana*.

The acute toxicity study on BAE did not produce any death in the animals at the highest dose used (5000 mg kg^−1^). There were also no signs of physical, neurological and autonomic toxicity. Therefore, the oral LD_50_ could be estimated to be above 5000 mg kg^−1^. The extract can thus be regarded as relatively non-toxic in mice since substances with an LD_50_ value of 1000 mg kg^−1^ by the oral route are regarded to have a low toxicity profile in subjects [23].

The acetic acid-induced abdominal writhing test is a very sensitive and convenient assay and provides a good analgesic profile for both peripheral and central acting chemicals [24]. Acetic acid produces nociception through peritoneovisceral inflammation, thus causing a decrease in systemic pH as well as release of endogenous pro-inflammogens such as prostanoids (PGE_2_ and PGF_2_), substance P, serotonin, histamine, sympathomimetic amines, bradykinin, serotonin, leukotrienes, cytokines (TNF-α, IL-1β and IL-8) that excite nociceptors [25]. The expression of these substances produces a spontaneous dorso-abdominal muscles contraction. The pain induced is easily attenuated by NSAIDS as well as opioids and other natural analgesics that act centrally [26]. The inhibitory effects of BAE on abdominal frequency and duration in this study, might be due to interference with central and peripheral transduction mechanisms by either decreasing release of the endogenous pro-inflammogens or interfering with the nociceptor activation by any of the inflammogens and/or reducing nociceptor sensitization to inflammogens action.

Oral administration of BAE reversed the muscle pain induced by intramuscular injection of a low pH saline solution into the gastrocnemius muscle of the rats. Two cycles of acidic saline injection is known to produce a non-inflammatory bilateral muscle mechanical hyperalgesia without any muscular activity impairment [27]. Previous reports suggest that, this mechanical hyperalgesia is reduced by anticonvulsant like Pregabalin and some opioid agonists [28, 29]. Acidic-saline stimulates the release of glutamate and aspartate and also causes an increase in NMDA and non-NMDA receptors activity. The nociceptive response induced by glutamate happens to engage peripheral, spinal and supraspinal mechanism as well as by the liberation of nitric oxide (NO) or by some NO-related substance [19, 28, 30]. Therefore, the suppression of the acidic saline-induced mechanical hyperalgesia by BAE is a complementary indication that the antinociceptive action of this extract could possibly be associated with the production and/or actions of glutamate, aspartate and NO. This is not surprising since there is a previous report on the inhibitory effects of *Burkea africana* extracts on production of NO [31, 32].

The formalin-induced nociception is a well-established *in vivo* model of acute pain and has been used in evaluating the analgesic potential of chemical substances in drug discovery [21]. The formalin test is characterized by two distinct phases of nociceptive response. The first phase (neurogenic phase) starts within seconds after formalin injection as a direct result of chemical activation of cationic peripherally localized transient receptor potential Ankyrin 1 (TRPA-1) receptors [33]. In the later phase (inflammatory phase), there is a release of inflammatory mediators such as prostaglandins, bradykinin, histamine, etc. and a great central sensitization of spinal circuit, secondary to the actions induced in primary afferents [21, 34]. This nociceptive model, therefore, can be employed to evaluate the anti-nociceptive as well as the possible mechanism of a suspected pain-relieving candidate [35,36]. BAE inhibited both phases of nociception in the formalin test but more effectively in the late inflammatory than the neurogenic phase.

An attempt to get more insight into the possible mechanisms involved in the observed anti-nociceptive effects of BAE revealed naloxone reversed the antinociceptive effects in both the neurogenic and inflammatory phases. This could be suggestive of a possible involvement of opioid receptors in the antinociceptive activity of BAE since naloxone is a non-selective opioid receptor antagonist with a greater affinity for the μ-receptor.

Serotonin (5-HT) receptors have been implicated in many disorders and 5-HT_3_ is prime candidates for antinociception, because of their functional diversity and their ability to mediate the release of neurotransmitters like dopamine, GABA, substance P and acetylcholine [37]. In the current study, granisetron inhibited the antinociceptive effects of BAE in both phases giving an indication that, 5-HT_3_ receptors may play critical roles in the observed actions of the extract. However, pretreatment with glibenclamie, nifedifine, yohimbine, theophylline or reserpine did not have any significant effect on the antinociceptive effects of BAE in the neurogenic phase but caused a partial reversal in the inflammatory phase.

## 5 Conclusions

The ethanolic stem bark extract of *Burkea africana* (BAE) is relatively non-toxic in mice with an LD_50_ above 5000 mg kg^−1^ and exerts central and peripheral antinociception effects with a possible involvement of 5-HT_3_ receptor and opioidergic pathways. However, further studies are needed isolates the active constituents and determine their exact mechanism of action.

## Declarations

### Author contribution statement

Yakubu Jibira: Conceived and designed the experiments; Performed the experiments; Analyzed and interpreted the data; Contributed reagents, materials, analysis tools or data; Wrote the paper.

Eric Boakye-Gyasi: Conceived and designed the experiments; Analyzed and interpreted the data; Contributed reagents, materials, analysis tools or data; Wrote the paper.

Wonder Kofi Mensah Abotsi: Analyzed and interpreted the data; Wrote the paper.

Isaac Kingsley Amponsah: Conceived and designed the experiments; Contributed reagents, materials, analysis tools or data; Wrote the paper.

Peter Duah; Frederick Kwadwo Baah: Performed the experiments.

Eric Woode: Conceived and designed the experiments; Analyzed and interpreted the data.

### Funding statement

This research did not receive any specific grant from funding agencies in the public, commercial, or not-for-profit sectors.

### Data availability statement

Data included in article/supplementary material/referenced in article.

### Declaration of interests statement

The authors declare no conflict of interest.

### Additional information

No additional information is available for this paper.
